# Pencil Graphite Electrocatalytic Sensors Modified by Pyrene Coated Reduced Graphene Oxide Decorated with Molybdenum Disulfide Nanoroses for Hydrazine and 4-Nitrophenol Detection in Real Water Samples

**DOI:** 10.3390/molecules28217311

**Published:** 2023-10-28

**Authors:** Alma Mejri, Giacomo Mandriota, Elfil Hamza, Maria Lucia Curri, Chiara Ingrosso, Abdelmoneim Mars

**Affiliations:** 1 Laboratory of Natural Water Treatment (LADVEN), Water Researches and Technologies Center, Techno-Park Borj-Cedria, University of Carthage, BP 273, Soliman 8020, Tunisia; 2 CNR-IPCF Sez. Bari, c/o Department of Chemistry, Università degli Studi di Bari, Via Orabona 4, I-70126 Bari, Italy; 3 Department of Chemistry, Università degli Studi di Bari, Via Orabona 4, I-70126 Bari, Italy

**Keywords:** Pencil Graphite Electrodes, Reduced Graphene Oxide, pyrene, MoS_2_ nanosheets, hydrazine, 4-nitrophenol, Differential Pulse Voltammetry, electrochemical sensors

## Abstract

Novel nanostructured platforms based on Pencil Graphite Electrodes (PGEs), modified with pyrene carboxylic acid (PCA) functionalized Reduced Graphene Oxide (rGO), and then decorated by chronoamperometry electrodeposition of MoS_2_ nanoroses (NRs) (MoS_2_NRs/PCA-rGO/PGEs) were manufactured for the electrocatalytic detection of hydrazine (N_2_H_4_) and 4-nitrophenol, pollutants highly hazardous for environment and human health. The surface morphology and chemistry of the MoS_2_NRs/PCA-rGO/PGEs were characterized by scanning electron microscopy (SEM), Raman, and X-ray photoelectron spectroscopy (XPS), assessing the coating of the PCA-rGO/PGEs by dense multilayers of NRs. N_2_H_4_ and 4-nitrophenol have been monitored by Differential Pulse Voltammetry (DPV), and the MoS_2_NRs/PCA-rGO/PGEs electroanalytical properties have been compared to the PGEs, as neat and modified by PCA-rGO. The MoS_2_NRs/PCA-rGO/PGEs demonstrated a higher electrochemical and electrocatalytic activity, due to their high surface area and conductivity, and very fast heterogeneous electron transfer kinetics at the interphase with the electrolyte. LODs lower than the U.S. EPA recommended concentration values in drinking water, namely 9.3 nM and 13.3 nM, were estimated for N_2_H_4_ and 4-nitrophenol, respectively and the MoS_2_NRs/PCA-rGO/PGEs showed good repeatability, reproducibility, storage stability, and selectivity. The effectiveness of the nanoplatforms for monitoring N_2_H_4_ and 4-nitrophenol in tap, river, and wastewater was addressed.

## 1. Introduction

Over the past years, the increased environmental contamination by toxic pollutants caused by industrialization, agriculture activities, and urban life, has raised a global concern for their harmful effects on human health and biodiversity, making urgent the need for sustainable development [1,2,3]. Globally, approximately 80% of industrial and municipal wastewater is discharged into the environment without any pre-treatment, and this situation has become a crucial concern in less developed countries, where there are no infrastructures for wastewater remediation [4]. Contaminations of rivers and wastewaters have constantly exposed people to toxic compounds causing numerous mental and physical dysfunctions, cancer, and weakening of the body’s immune system, lowering life expectancy, and resulting, in many cases, in mortality [5]. Around 829,000.00 people, including 300,000.00 children under five years old, die every year from diseases resulting from lack of hygiene and polluted drinking water [6].

In this frame, the manufacturing of innovative and sustainable solutions, user-friendly and cost-effective, for reliable quality control of the waters, is increasingly in demand.

Hydrazine (N_2_H_4_) is among the most dangerous pollutants because its toxicity can generate irreversible cell damage, and develop complications such as brain and liver dysfunction, DNA damage, and leukemia. Despite this, N_2_H_4_ has numerous uses as a reducing agent, emulsifier, catalyst, antioxidant, corrosion inhibitor, and as a precursor of explosives, dyestuffs, pesticides, herbicides, insecticides, and pharmaceutical derivatives. U.S. Environmental Protection Agency (EPA) has classified it as a potent carcinogen, with a recommended level in drinking water lower than 10 ppb [7]. 4-nitrophenol (4-NP) is another potential carcinogen and mutagenic agent that causes acute effects such as headache, nausea, drowsiness, cyanosis, and cancer. 4-NP is used in industries of synthesis of drugs, leather processing, dye synthesis, and preparation of organo-phosphorus pesticides, such as methyl parathion and ethyl parathion, although it is in the “Priority Pollutant List” of U.S. EPA, with a recommended upper limit in drinking water of 10 ppb [8].

Among the conventional analytical technologies used for monitoring N_2_H_4_ and 4-NP, there are gas chromatography/mass spectrometry (GC/MS), atomic absorption spectroscopy (AAS), high-performance liquid chromatography (HPLC), spectrofluorimetry, capillary electrophoresis, and flow injection chemiluminescence [9,10]. Such analytical tools are laborious, expensive, require advanced skills for their operation, and are difficult to install for the bulky size of their devices. By contrast, electrochemical sensors offer the advantage of the rapidity of analysis and cost-effectiveness, and their reduced size makes them portable and usable on-site [11]. Electrode modification is the strategy used to improve the sensitivity and selectivity of these sensors, reproducibility of the electrode surface behavior, and accelerate the kinetics of the electrochemical reactions of several compounds [12].

Graphene-based nanostructures have found application in this type of sensor for their high electrochemical stability, high electrocatalytic activity, and fast heterogeneous electron transfer kinetics [13]. Also, 2D layer-structured transition-metal dichalcogenide nanomaterials, such as molybdenum disulfide (MoS_2_) semiconductors, have attracted attention for their interesting electric tunable properties, depending on crystalline structure, nanosheet size, and structural surface defects [14]. The preparation of hybrid nanocomposites based on graphene derivatives decorated with nanostructured MoS_2_ results in materials showing enhanced stability, electron conductivity, heterogeneous electron transfer kinetics, and electrocatalytic activity [15].

Herein, hybrid nanocomposite-modified nanoplatforms formed by pencil graphite electrodes (PGEs) coated by Reduced Graphene Oxide (rGO) sheets, functionalized with 1-pyrene carboxylic acid (PCA), then decorated by chronoamperometry electrodeposition, with a dense layer of MoS_2_ nano roses (NRs), have been investigated for the electrocatalytic detection of N_2_H_4_ and 4-NP by Differential Pulse Voltammetry (DPV).

1-pyrene carboxylic acid (PCA) has been used to allow liquid phase exfoliation of rGO [16], as it acts as a linker for binding the rGO basal plane by π-π interactions and the MoS_2_ NRs by its carboxyl functionalities, and to promote NRs-rGO electron coupling, providing effective merging of functionalities of the two materials [17].

PGE-based sensors have been selected, because are a practical and not expensive sensing technology, rapid, compact, and suited for portable use and for on-site monitoring.

The manufactured MoS_2_NRs/PCA-rGO/PGEs have shown a LOD for N_2_H_4_ and 4-NP of 9.3 nM and 13.3 nM, respectively lower than the U.S. EPA recommended concentration in drinking water and comparable with the lowest ones reported [18,19,20,21,22,23], with values of repeatability, reproducibility, storage stability, and selectivity, suited for monitoring the selected hazardous, in tap, river, and wastewater samples.

## 2. Results and Discussion

### 2.1. Decoration of the PCA-rGO/PGEs with MoS_2_ NRs and Characterization

**Figure 1:**
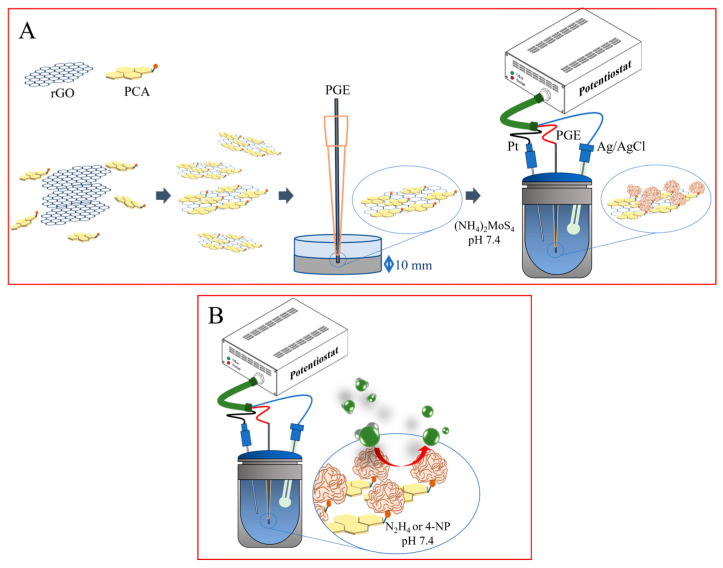
Scheme of (**A**) exfoliation and functionalization of rGO with PCA, deposition of rGO onto PGEs and electrochemical deposition of MoS_2_ NRs onto PCA-rGO/PGEs, and (**B**) electrochemical analytes detection.

The MoS_2_NRs/PCA-rGO/PGEs were manufactured starting from the liquid phase exfoliation [16] of rGO with 1-pyrene carboxylic acid (PCA) (Figure 1A), which binds by π-π interactions the rGO basal plane and the by the carboxyl functionalities the MoS_2_ NRs, enabling NRs-rGO electron coupling [17]. Then, the PCA-rGO/PGEs were prepared by dipping the PGEs in an ethanol dispersion of PCA-rGO, and subsequently electrodepositing the MoS_2_ NRs by chronoamperometry, after dipping in a 5 mM (NH_4_)_2_MoS_4_ precursor solution at pH 7.4 (Figure 1A).

The effectiveness of the chronoamperometry electrodeposition of the MoS_2_ NRs was investigated by registering the reduction current of the (NH_4_)_2_MoS_4_ precursor solution at different pH and changing the deposition time (Figure S1 of the Supplementary Information) to determine the most suited experimental conditions for achieving the highest reduction current, that was indeed obtained at pH 7.4 (Figure S1A) and after 90 s of electrodeposition (Figure S1B).

Scanning Electron microscopy (SEM), Raman, and X-ray Photoelectron Spectroscopy (XPS) investigation were carried out to study surface morphology and chemistry of the PGEs, as neat, and after deposition of PCA-rGO and electrodeposition of the MoS_2_ NRs.

**Figure 2:**
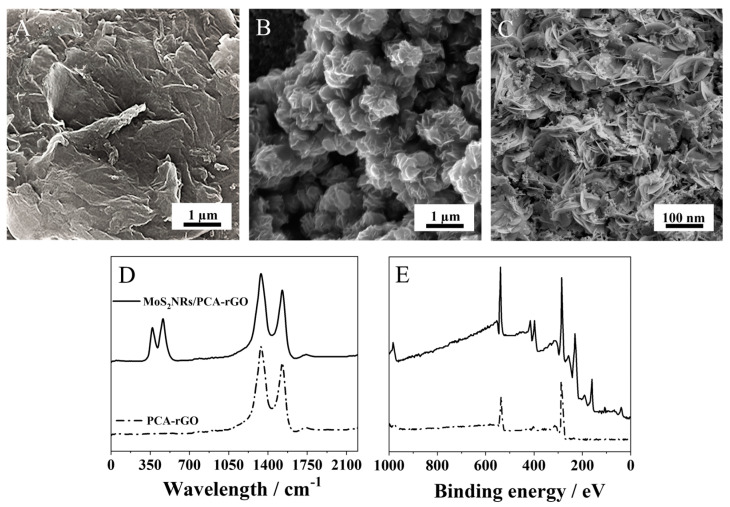
SEM images of (**A**) PCA-rGO/PGEs (21 KX) and (**B**,**C**) MoS_2_NRs/PCA-rGO/PGEs (28.5 KX (**B**), 220 KX (**C**)). (**D**) Raman and (**E**) XPS survey spectra of the PCA-rGO/PGEs (dash-dot line) and MoS_2_NRs/PCA-rGO/PGEs (solid line).

The PGEs show the porous surface morphology (Figure S2A) of the graphite texture. After dipping in the PCA-rGO dispersion, the electrodes are coated by sheets-like structures recalling the typical surface morphology of the PCA-rGO sheets (Figure S2B), which appear almost smooth on the surface featuring bright wrinkles ascribed to folded edges and mechanical lattice deformations (Figure 2A). After electrodeposition of (NH_4_)_2_MoS_4_, the PCA-rGO/PGEs surface morphology is characterized by a coating formed by a multilayer of nanoroses (NRs)-like structures (Figure 2B), generated by the assembling of MoS_2_ nanosheets (Figure 2C), as demonstrated elsewhere [24].

The comparison of the Raman spectra of the MoS_2_NRs/PCA-rGO/PGEs and PCA-rGO/PGEs shows the same Raman modes, namely the D peak at 1335 cm^−1^, originating from the breathing modes of C-sp^2^ atoms in hexagonal rings, and the G peak, which is due to the bond stretching between two C-sp^2^ atoms at ca. 1530 cm^−1^ [23] (Figure 2D). The intensity ratio between the D and G peaks of rGO is almost preserved after NRs electrodeposition (Figure 2D) and is typically used as an indication of the graphitic material structural quality [25], such a result addresses NRs formation with preservation of the rGO structure. These spectra also show peaks at 370 cm^−1^ and 465 cm^−1^ attributed to the E^1^_2g_ and A_1g_ modes of the MoS_2_ NRs [26], assessing their effective electrodeposition onto the PCA-rGO/PGEs (Figure 2D).

XPS survey spectra of the MoS_2_NRs/PCA-rGO/PGEs further show the electrode change of chemistry, with the typical O1s and C1s components of PCA-RGO at 540.33 eV and 287.32 eV, respectively, and the Mo3p, Mo3d, and S2p components of the MoS_2_ NRs at 399.53, 232.32 and 161.92 eV [27], respectively (Figure 2E). In the spectra of the MoS_2_NRs/PCA-rGO/PGEs, the O1s and C1s components are shifted of ca. 2.2 eV with respect to the PCA-rGO/PGEs counterparts, which are at 538.12 eV and 289.52 eV, respectively (Figure 2E), attesting a change of the electron densities of the O and C atoms of PCA-RGO, ascribed to the binding, by coordination, of the MoS_2_ NRs [24], mediated by PCA [25,26,27].

**Figure 3:**
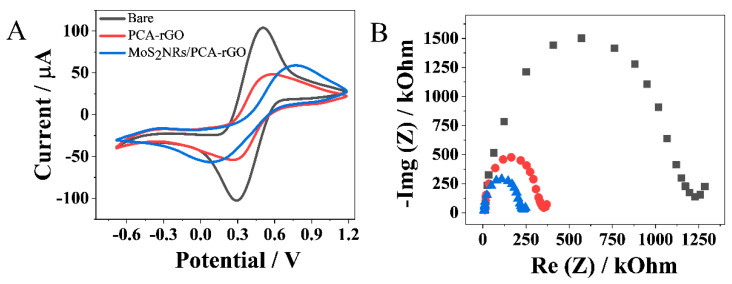
(**A**) CV scans at 50 mV s^−1^ and (**B**) EIS spectra of neat PGEs, PCA-rGO/PGEs, and MoS_2_NRs/PCA-rGO/PGEs, in 0.01 M PBS added with 0.1 M KCl and 5 mM Fe[(CN)_6_]^3−/4−^ (pH 7.4).

The electrodeposition of the MoS_2_ NRs onto PCA-rGO was studied by Cyclic Voltammetry (CV) and Electrochemical Impedance Spectroscopy (EIS) (Figure 3) using the Fe[(CN)_6_]^3−/4−^ probe.

The CV curves recorded at the MoS_2_NRs/PCA-rGO/PGEs and PCA-rGO/PGEs show a decrease in the anodic and cathodic peak potentials difference (ΔEp) with respect to PGEs, which show the typical quasi-reversible redox peaks of [Fe(CN)_6_]^3−/4−^ (Figure 3A). This result shows higher reversibility of the probe at both the modified electrodes, which is accounted for by their higher conductivity and higher electron transfer capability with the electrolyte, as demonstrated by the increase in their K_0_ with respect to the PGEs (Table 1). In particular, the decrease in ΔEp is higher at the MoS_2_NRs/PCA-rGO/PGEs than at the PCA-rGO/PGEs, (Figure 3A) for (i) occurrence of MoS_2_NRs-rGO electron coupling interactions mediated by PCA that are responsible for the increase in the electrode conductivity [17,18,19,20,21,22,23,24,25,26,27,28,29], and (ii) the catalytic activity of the MoS_2_ NRs that favors the [Fe(CN)_6_]^3−/4−^ red/ox reactions at the electrode [15]. Besides, PCA increases the electric conductivity of the PCA-rGO/PGEs acting as electrical “glue” among rGO sheets, [30] and bears oxygen-containing moieties undergoing red/ox reactions, [31] that increase K_0_ of the PCA-rGO/PGEs.

Finally, a significant increase in the anodic current is observed at the MoS_2_NRs/PCA-rGO/PGEs (Figure 3A), likely for their higher A_ele_ (Table 1), assessing their higher electrochemical activity. This evidence is supported also by the Faradaic impedance spectra of Figure 3B, which show, for the MoS_2_NRs/PCA-rGO/PGEs, a reduction of the semicircle diameter, demonstrating a decrease in R_et_ (Table 1), which confirms the higher reversibility of the probe at such electrodes.

### 2.2. Electroanalytical Investigation of the MoS_2_ NRs/PCA-rGO/PGEs

#### 2.2.1. Electrochemical Detection of N_2_H_4_ and 4-NP at the MoS_2_NRs/PCA-rGO/PGEs

The manufactured MoS_2_NRs/PCA-rGO/PGEs were studied for the detection of N_2_H_4_ and 4-NP, respectively, by cyclic voltammetry (CV) in a three-electrode cell (Figure 1B). At first, the electrode response to the selected analytes was investigated in the range of pH of the (NH_4_)_2_MoS_4_ precursor solution and of the analyte solutions between 3 and 9.5. The results show that the higher electrocatalytic activity and sensitivity of the MoS_2_NRs/PCA-rGO/PGEs were achieved with (NH_4_)_2_MoS_4_ (Figure S3A) and analytes solutions at pH 7.4 (Figure S3B).

**Figure 4:**
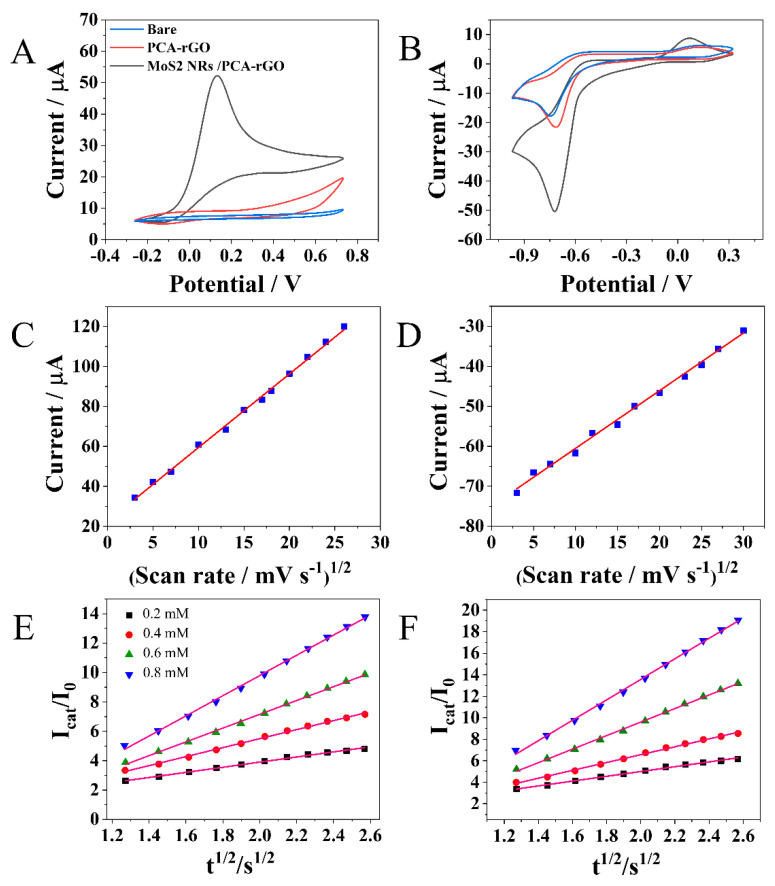
CVs collected at the PGEs, PCA-rGO/PGEs and MoS_2_NRs/PCA-rGO/PGEs, at 50 mV s^−1^, in 0.1 mM PBS buffer (7.4 pH), (**A**) 1 mM in N_2_H_4_ from −0.3 V to 0.7 V, and (**B**) 1 mM in 4-NP from −1 V to 0.3 V. Oxidation currents of N_2_H_4_ at 0.13 V (**C**,**E**) and reduction currents of 4-NP at −0.71 V (**D**,**F**) at the MoS_2_NRs/PCA-rGO/PGEs, between 100–500 mV s^−1^, versus square root of scan rate (**C**,**D**) and versus square root of time (**E**,**F**), in the 0.2 mM–0.8 mM range, in 0.1 M PBS buffer solutions (pH 7.4).

The CV curves collected at the PGEs and PCA-rGO/PGEs in the presence of N_2_H_4_ do not present any cathodic peak. Conversely, the MoS_2_NRs/PCA-rGO/PGEs show an intense peak at 0.13 V (vs. Ag/AgCl, saturated KCl) (Figure 4A) accounted for by the N_2_H_4_ oxidation catalyzed by MoS_2_ NRs [32], that leads to formation of N_2_H_4_^+^ then oxidized to N_2_ [33]. The lack of a peak in the cathodic sweep of N_2_H_4_ addresses the irreversibility of the oxidation process (Figure 4A) [33].

The CVs collected at the PGEs, in PBS buffer solution added with 4-NP, show its reduction peak to 4-aminophenol [34] at ca. −0.75 V (vs. Ag/AgCl, saturated KCl) (Figure 4B), that shifts to −0.73 V (vs. Ag/AgCl, saturated KCl) at the PCA-rGO/PGEs, and further to −0.71 V (vs. Ag/AgCl, saturated KCl) at the MoS_2_ NRs/PCA-rGO/PGEs (Figure 4B), demonstrating that the reduction is more energetically favored at the MoS_2_ NRs/PCA-rGO/PGEs for their higher conductivity and higher K_0_ (Table 1).

Besides, the increase in the current intensity at the PCA-rGO/PGEs and MoS_2_NRs/PCA-rGO/PGEs is accounted for by the enhancement of their A_ele_ (Table 1).

Finally, in Figure 4B, a peak between −0.1–0.3 V, due to the oxidation of 4-quinoimine, the reduction product of 4-aminophenol [34], is evident. Such a peak is more intense and shifts toward lower potential values at the MoS_2_NRs/PCA-rGO/PGEs (Figure 4B) for the electrocatalytic properties of the NRs [15].

The trend of the current intensity against the square root of scan rate (v^1/2^) of N_2_H_4_ and 4-NP was collected to study the charge transport across the MoS_2_NRs/PCA-rGO/PGEs and mass transfer regime (Figure 4C,D). The oxidation current of N_2_H_4_ and the reduction current of 4-NP can be fitted by linear regression, with a correlation coefficient of 0.99, increasing linearly with the increase in v^1/2^ (Figure 4C,D). These results assess the occurrence of diffusion-controlled electron transfers.

The K_cat_ values of the N_2_H_4_ oxidation and 4-NP reduction at 0.13 V (vs. Ag/AgCl, saturated KCl) and −0.71 V (vs. Ag/AgCl, saturated KCl), respectively were estimated at the MoS_2_NRs/PCA-rGO/PGEs by chronoamperometry, plotting their catalytic and initial current intensity ratio (I_cat_/I_0_) versus the square root of time (t^1/2^), respectively, between 0.2 mM–0.8 mM (Figure 4E,F).

The results present a linear relationship of I_cat_/I_0_ vs. t^1/2^, and K_cat_ values of 7.1 mM^−1^ s^−1^ for N_2_H_4_ and 6.2 mM^−1^ s^−1^ for 4-NP, attesting to the high electrocatalytic activity of the MoS_2_NRs/PCA-rGO/PGEs.

#### 2.2.2. Determination of LOD, Repeatability, Reproducibility, and Storage Stability of MoS_2_NRs/PCA-rGO/PGEs and Interference Effects

**Figure 5:**
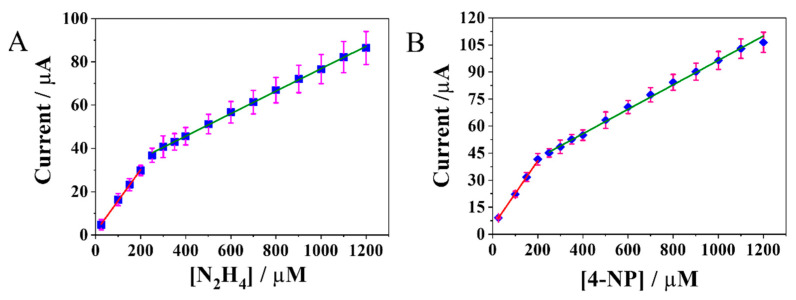
Calibration plots of 25 µM–1200 µM N_2_H_4_ (**A**) and 4-NP (**B**), in 0.1 M PBS buffer (pH 7.4) at the MoS_2_ NRs/PCA-rGO/PGEs, with 0.05 s modulation time, 0.2 s interval time, 60 mV modulation amplitude, 10.5 mV step potential and 50 mV s^−1^ scan rate.

Differential Pulse Voltammetry (DPV) investigation was carried out at the MoS_2_ NRs/PCA-rGO/PGEs in N_2_H_4_ and 4-NP standard solutions, respectively, in the concentration range of 25 µM–1200 µM, to evaluate their electrocatalytic properties and collect calibration curves (Figure 5).

The DPV curves present an increase in the electrocatalytic current with the enhancement of the analyte concentration, showing a linear relationship of type (y = (a ± b)x + c ± d) with a correlation coefficient of r^2^ = x. Besides, both the calibration plots have two different slopes (Figure 5), demonstrating two different electrocatalytic kinetic processes, that depend on the analyte concentration, and are likely ascribed to a change of the electrode surface chemistry induced by the red/ox reactions [35]. At low concentrations, the electrocatalytic processes evolve by analyte adsorption at the electrode surface active sites, providing a high sensitivity. At higher concentrations, the surface sites are partially saturated, and hence, the activation step of the analyte in the red/ox reaction is slowed down, becoming the rate-determining step that decreases sensitivity [35].

The sensitivity (S), limit of detection (LOD), limit of quantification (LOQ), % RSD of repeatability, % RSD of reproducibility, and storage stability of the MoS_2_NRs/PCA-rGO/PGEs were evaluated in the detection of N_2_H_4_ and 4-NP, respectively (Table 2).

Interestingly, the determined LODs are lower than the U.S. EPA recommended concentration thresholds of the analytes [7,8] and comparable with the LODs reported for state-of-the-art sensors [18,19,20,21,22,23] (Table S1).

Chronoamperometric measurements were carried out to investigate repeatability, reproducibility, and stability of the MoS_2_NRs/PCA-rGO/PGEs for N_2_H_4_ and 4-NP detection at +0.13 V and −0.71 V (vs. Ag/AgCl, saturated KCl), respectively.

Repeatability was investigated by measuring the electrocatalytic current of N_2_H_4_ and 4-NP nine times in one day (Figure S4) at the same electrode, and % RSD of 3.3 and 3.6, respectively were estimated (Table 2), as shown by Figures S5A and S6A. Reproducibility was assessed using nine hybrid platforms (Figure S7), and % RSD of 3.4 and 3.7 (Table 2), respectively were found as evidenced by Figures S5B and S6B. The storage stability was determined over a period of one month, monitoring the electrocatalytic currents of nine MoS_2_NRs/PCA-rGO/PGEs stored at 4 °C, every week (Figure S8), revealing almost stable values (Table 2), as shown by Figures S5C and S6C.

Matrix components can detrimentally affect the LOD, LOQ, repeatability, and reproducibility of the measurements. For this reason, the selectivity of the MoS_2_NRs/PCA-rGO/PGEs was tested in the presence of the typical interferents of N_2_H_4_ and 4-NP. Citric acid, uric acid, ethanol, and glucose were chosen to test the selectivity towards N_2_H_4_, whilst catechol, hydroquinone, and 2,4-dinitrobenzene for 4-NP (Figure S9). DPV curves of the MoS_2_NRs/PCA-rGO/PGEs were recorded in 0.1 M PBS solutions (pH 7.4) and 0.8 mM in N_2_H_4_ and 4-NP, respectively, separately spiked with 100-fold more concentrated interfering species. The results show that, although added at a 100-fold higher concentration, the interferent species do not significantly affect the current intensities (Figure S9) and show a % RSD of 3.7% for both the analytes, demonstrating a high electrode selectivity.

#### 2.2.3. Quantification of N_2_H_4_ and 4-NP in Real Samples

The effectiveness of the MoS_2_NRs/PCA-rGO/PGEs in the determination of N_2_H_4_ and 4-NP in river, tap, and wastewater, was studied by chronoamperometry (Figure S10), performing the analyses by the standard addition method, as described in the experimental section, and comparing the results with those obtained from HPLC analyses (Table 3).

As shown in Table 3, the achieved recovery rates demonstrate the reliability of the MoS_2_NRs/PCA-rGO/PGEs in the detection of the selected analytes in real water samples.

## 3. Materials and Methods

### 3.1. Reagents and Instrumentation

Commercial Reduced Graphene Oxide (rGO) (1.6 nm thick flakes) was purchased from Graphene Supermarket. 1-Pyrene Carboxylic Acid (PCA, 97%), ammonium tetrathiomolybdate ((NH_4_)_2_MoS_4_, 99.97%), potassium chloride, phosphate saline buffer (PBS) tablets, ferricyanide (Fe[CN)_6_]^3−^) and ferrocyanide (Fe[CN)_6_]^4−^), sulfuric acid (H_2_SO_4_, 96%) ethanol, toluene, hydrazine solution (N_2_H_4_, 35 wt% in water), 4-nitrophenol (4-NP), citric acid, uric acid, glucose, catechol, hydroquinone and 2,4-dinitrobenzene were obtained from Sigma Aldrich (Merck KGaA, Darmstadt, Germany). All the solutions were prepared with Milli-Q water. 0.7 mm micro carbon 250 2H graphitic pencils, from STAEDTLER Mars (Nuernberg, Germany), were from a local market.

Raman spectra were collected by a LabRAM HR Evolution spectrophotometer from HORIBA, equipped with a 100× microscope objective lens and a continuous excitation laser diode at 532 nm.

X-ray Photoelectron Spectroscopy (XPS, Kratos Axis Ultra) was performed by a monochromatic Al Ka source (at 1486.58 eV), operating with a spot size of 200 µm, at a take-off angle of 70°. Survey (0–1000 eV) spectra were collected at a pass energy of 160 eV. Charge correction of the spectra was performed considering the sp^2^ carbon component of the C1s spectrum as an internal reference (Binding Energy, BE = 30 eV).

Scanning Electron Microscopy (SEM) images were recorded by a Zeiss Sigma microscope, equipped with both an in-lens secondary electron and an INCA Energy Dispersive Spectroscopy (EDS) detector. Samples were fixed onto stainless-steel holders by using carbon tape.

High-performance liquid chromatography (HPLC) analyses were performed by an Agilent 1100 HPLC analyzer (Santa Clara, CA, USA) by using a C18 column (5 µm, 4.6 mm × 250 mm) from Waters (Milford, MA, USA), equipped with an absorption spectrophotometer.

Cyclic Voltammetry (CV), Differential Pulse Voltammetry (DPV), Chronoamperometry, and Electrochemical Impedance Spectroscopy (EIS) measurements were performed by a Metrohm Autolab PGSTAT 302n electrochemical workstation (Herisau, Switzerland), equipped with the Nova^®^ v1.11 software and a three-electrode cell, where the pencil graphite electrode (PGE), a platinum wire and an Ag/AgCl (3 M KCl) electrode are the working, counter, and reference electrodes, respectively (Figure 1). The electrical connection between the electrochemical workstation and the PGEs was settled welding a copper wire onto the metallic holder of the graphite pencil.

### 3.2. Exfoliation and Functionalization of Reduced Graphene Oxide (rGO) with 1-Pyrene Carboxylic Acid (PCA)

PCA-rGO was prepared by exfoliating and functionalizing commercial rGO with PCA following a reported procedure with minor modifications [36], stirring and sonicating a mixture of PCA and rGO powders prepared in a 17:1 *w*/*w* in n-methyl-2-pyrrolidone (NMP), in an ice-cooled bath. Centrifugation cycles (9000 rpm for 20 min) and re-dispersion in methanol were carried out to remove PCA in excess. The purified PCA-rGO complex is formed of flakes of single and few-layer graphene and multi-layer graphene [16,17].

### 3.3. Modification of PGEs with PCA-rGO and Decoration with MoS_2_ Nanoroses

PGEs were polished with a weighing paper to achieve an almost flat surface and then were sonicated in a 1 M H_2_SO_4_ solution, for 2 min, to graft oxygen-based groups, leading to a significant increase in the PGE electrochemical reactivity [37].

The PCA-rGO modified PGEs (PCA-rGO/PGEs) were obtained by soaking 10 mm of a 0.7 mm 2H graphitic pencil, into a 2.5 mg mL^−1^ PCA-rGO dispersion in ethanol for 30 min. In this step, PCA-rGO binds the H_2_SO_4_-treated graphitic electrode by π-π stacking forces and hydrogen bond interactions.

MoS_2_ nanoroses (NRs) were electrodeposited onto the PCA-rGO/PGEs by chronoamperometry, by dipping 10 mm of the PCA-rGO/PGEs in a 5 mM (NH_4_)_2_MoS_4_ precursor solution, 0.1 M in KCl, and applying, for 90 s, a potential of −1 V (vs. Ag/AgCl, saturated KCl), that is the reduction potential of MoS_4_^2−^.

### 3.4. Electrochemical Investigation of the MoS_2_ NRs/PCA-rGO/PGEs

The functionalization of the PGEs with PCA-rGO and decoration with MoS_2_ NRs were studied by cyclic voltammetry (CV) and electrochemical impedance spectroscopy (EIS), in a 0.01 M PBS buffer solution added with 0.1 M KCl and 5 mM Fe[(CN)_6_]^3−/4−^ at pH 7.4. CV scans were collected at the 50 mV s^−1^ scan rate.

Faradaic impedance spectra were reported as Nyquist plots, and the collected data were treated by Randles equivalent circuits by the Nova^®^ v1.11 software to estimate charge transfer resistance (R_et_).

Electroactive surface area (A_ele_) was calculated by the Randles-Sevcik equation for a quasi-reversible system, as:

I_ap_ = (2.69 × 10^5^) A_ele_ × C × D^1/2^ × n^3/2^ × v^1/2^ (1)

where I_ap_ is the anodic peak current, n the number of electrons transferred, D the [Fe(CN)_6_]^4−^ diffusion coefficient equal to 6.5 × 10^−6^ cm^2^ s^−1^, v the potential scan rate (V s^−1^) and *C* the [Fe(CN)_6_]^4−^ concentration (mol cm^−3^).

The heterogeneous electron transfer rate constant (k_0_) was determined as:

k_0_ = R/(n^2^ × F^2^ × A_ele_ × C × R_et_) (2)

where R is the universal gas constant and F is the Faraday constant.

Electrocatalytic rate constants (K_cat_) were calculated by chronoamperometry in 0.1 M PBS buffer solutions at pH 7.4, 1 mM in N_2_H_4_ and 4-NP, respectively, by using the Cottrell equation as [38]:

I_cat_/I_0_ = (π × K_cat_ × C × t)^1/2^ (3)

where I_cat_ and I_0_ are the currents collected with and without the analyte, respectively at the concentration C, K_cat_ is the electrocatalytic rate constant, and t is the measurement time.

### 3.5. Electroanalytical Application

N_2_H_4_ and 4-NP were monitored by differential pulse voltammetry (DPV) dipping the PGEs for 10 mm into a 0.1 M PBS buffer solution at pH 7.4, added by N_2_H_4_ and 4-NP, in the concentration range of 20–1200 µM, respectively, with modulation time of 0.05 s, interval time of 0.2 s, modulation amplitude of 60 mV, step potential of 10.5 mV and scan rate of 50 mV s^−1^, with an electrode geometrical area of 44.4 mm^2^.

Calibration plots were fitted to a linear model function (y = ax +b) by the weighted linear least squares method by using Origin Pro 2018 (Origin 8 V8.0951(B951)) and w = 1/σ_i_ ^2^, as weight.

The Limit of detection (LOD) was calculated as

LOD = 3.3 (s_y/x_/S) (4)

where s_y/x_ is the residual standard deviation and S is the slope of the calibration plot (calibration sensitivity) [39].

The limit of quantification (LOQ) was determined as

LOQ = 10 (s_y/x_/S) (5)

Chronoamperometry was recorded at the oxidation potential of N_2_H_4_ or reduction potential of 4-NP, namely 0.13 V (vs. Ag/AgCl, saturated KCl) and −0.71 V (vs. Ag/AgCl, saturated KCl), respectively to determine K_cat_, as well as repeatability, reproducibility, and stability of MoS_2_NRs/PCA-rGO/PGEs for the determination of N_2_H_4_ and 4-NP.

Real samples of Tunis tap water and Majerda river and wastewater, treated by a 0.2 µm PTFE filter membrane to eliminate suspended particles and adjusted to pH 7.4, were used to detect N_2_H_4_ and 4-NP separately.

In the calibration plots, mean values of the analyte concentrations were determined by chronoamperometry by the standard addition method, analyzing three aliquots of real water samples spiked by N_2_H4 and 4-NP, in the concentration ranges between 300–900 µM and 400–1000 µM, respectively. Errors in both variables (X, Y) were determined by using the linear “errors-in-variables regression method”. The mean concentrations were evaluated against those achieved by high-performance liquid chromatography (HPLC), the approach conventionally used to determine N_2_H_4_ and 4-NP in real water samples [40].

## 4. Conclusions

A nanostructured platform formed of Pencil Graphite Electrodes (PGEs) modified with a hybrid nanocomposite based on 1-pyrene-carboxylic acid functionalized RGO (PCA-rGO) sheets, decorated with electrodeposited MoS_2_ nano roses (NRs) (MoS_2_NRs/PCA-rGO/PGEs), was prepared for the electrochemical determination of the highly toxic N_2_H_4_ and 4-nitrophenol (4-NP) pollutants.

The MoS_2_NRs/PCA-rGO/PGEs demonstrated an electrocatalytic activity higher than the PCA-rGO/PGEs and PGEs, due to their higher electroactive surface area and electric conductivity, and to the electrocatalytic properties of the MoS_2_ NRs.

LODs of 9.3 and 13.7 nM were estimated for the detection of N_2_H_4_ and 4-NP, respectively, concentration values lower than those recommended by the U.S. EPA for drinking water, and in line with the lowest ones found in state-of-the-art MoS_2_/graphene nanocomposites. The MoS_2_NRs/PCA-rGO/PGEs showed a % RSD of repeatability and reproducibility of 3.3 and 3.6, and 3.4 and 3.7, respectively for N_2_H_4_ and 4-NP, and a storage stability that decreases by 9.1% and 12.6% within a month. A high selectivity with a % RSD of 3.1 and 3.5 for N_2_H_4_ and 4-NP, respectively was found in the presence of interferent species at concentrations 100-fold higher than those of the analytes. Effective reliability of the prepared nanoplatforms for monitoring the selected pollutants in real water samples was assessed by the recovery rate values, which were found between 99.3–101.3% and 98.17–100.6%, respectively, envisioning their applicability in monitoring other toxic species.

## Figures and Tables

**Table 1:** Electroactive surface area (A_ele_), apparent heterogeneous electron transfer constant (K_0_) and electron transfer resistance (R_et_) of PGEs, PCA-rGO/PGEs and MoS_2_NRs/PCA-rGO/PGEs.

	A_ele_/mm^2^	K_0_/cm s^−1^	R_et_/kOhm
Bare PGE	1.25 ± 0.03	0.004 ± 0.021	1193.0 ± 10.7
PCA–rGO/PGE	1.01 ± 0.02	0.006 ± 0.034	355.0 ± 4.9
MoS_2_NRs/PCA-rGO/PGE	2.19 ± 0.04	0.013 ± 0.042	237.0 ± 8.2

**Table 2:** Sensitivity (S), Limit of Detection (LOD), Limit of Quantification (LOQ), and % RSD of repeatability and reproducibility and storage stability of the MoS_2_NRs/PCA-rGO/PGEs towards 0.5 mM N_2_H_4_ and 0.5 mM 4-NP, respectively.

	N_2_H_4_	4-NP
Sensitivities/mA mM^−1^	0.051	0.054
LOD/nM	9.3	13.7
LOQ/nM	30.51	45.34
%RSD of repeatability	3.3	3.6
%RSD of reproducibility	3.4	3.7
Storage stability	Decrease by 9.1%	Decrease by 12.6%

**Table 3:** Determination of N_2_H_4_ and 4-NP in tap, river, and wastewater.

Samples	Analytes	Standard Concentration (mM)	Concentration Determined by Chronoamperometry (mM)	Recovery Rate % Determined by Chronoamperometry	Concentration Determined by HPLC (mM)	Recovery Rate % Determined by HPLC
Tap water	N_2_H_4_	300	298.2	99.4	299.7	99.9
		500	497.4	99.5	500.7	100.1
		800	802.1	100.3	801.3	100.2
River water		400	397.2	99.3	400.7	100.2
		500	501.7	100.3	499.7	99.9
		600	601.8	100.3	599.3	99.9
Wastewater		500	504.7	100.9	501.4	100.3
		700	702.9	100.4	703.1	100.4
		900	912.1	101.3	907.3	100.8
Tap water	4-NP	400	400.6	100.1	401.1	100.3
		600	596.7	99.5	601.9	100.3
		800	798.5	99.8	799.2	99.9
River water		400	400.3	100.1	398.6	99.6
		500	493.7	98.7	501.3	100.3
		600	599.2	99.9	601.2	100.2
Wastewater		400	392.7	98.2	400.8	100.2
		700	704.2	100.6	701.2	100.1
		1000	989.6	99.0	996.3	99.6

## Data Availability

Data sharing not applicable.

## Supplementary Materials

The following supporting information can be downloaded at: https://www.mdpi.com/article/10.3390/molecules28217311/s1, Figure S1: Optimization of the chronoamperometry electrodeposition of the MoS_2_ NRs onto the PCA-rGO/PGEs. Figure S2: SEM image of PGEs and (B) TEM image of PCA-rGO. Figure S3: Current response of the MoS_2_NRs/PCA-RGO/PGEs towards N_2_H4 and 4-NP, respectively at different pH of the (NH_4_)_2_MoS_4_ solutions and of the N_2_H4 and 4-NP solutions, ranging between 3.5 and 9. Table S1: LODs of state-of-the-art sensors for N_2_H_4_ and 4-NP detection. Figure S4: Chronoamperograms of repeatability tests of N_2_H_4_ and 4-NP detection. Figure S5: Histograms of repeatability, reproducibility, storage stability, and selectivity tests for the detection of N_2_H_4_ at the MoS_2_NRs/PCA-rGO/PGEs. Figure S6: Histograms of repeatability, reproducibility, storage stability, and selectivity tests for the determination of 4-NP at the MoS_2_NRs/PCA-rGO/PGEs. Figure S7: Chronoamperograms of reproducibility tests of N_2_H_4_ and 4-NP at the MoS_2_NSs/PCA-rGO/PGEs. Figure S8: DPVs of N_2_H_4_ and 4-NP at the MoS_2_NSs/PCA-rGO/PGEs after one, two, three, and four weeks. Figure S9: DPVs of N_2_H_4_ and 4-NP at the MoS_2_NSs/PCA-rGO/PGEs in the presence of interfering species. Figure S10: Chronoamperograms at the MoS_2_NRs/PCA-rGO/PGEs for the detection of N_2_H_4_ and 4-NP, in river, tap, and wastewater samples, respectively.
