# Supplementary material for: Pencil Graphite Electrocatalytic Sensors Modified by Pyrene Coated Reduced Graphene Oxide Decorated with Molybdenum Disulfide Nanoroses for Hydrazine and 4-Nitrophenol Detection in Real Water Samples
Source: Molecules. 2023 Oct 28;28(21):7311. doi: 10.3390/molecules28217311 (PMC10648802; doi:10.3390/molecules28217311)
Supplement: Supplementary file 1 [file molecules-28-07311-s001.zip › molecules-2677455-supplementary.pdf]

Supplementary Materials

Pencil graphite electrocatalytic sensors formed by Pyrene coated Reduced Graphene Oxide decorated with molybdenum disulfide nanosheets for hydrazine and 4-nitrophenol detection in real water samples

Alma Mejri[1], Giacomo Mandriota[2], Hamza Elfil[1], Maria Lucia Curri[2,3], Chiara Ingrosso[*,2], Abdelmoneim Mars[1,*]

[1]Laboratory of Natural Water Treatment (LADVEN), Water Researches and Technologies Center, Techno-park Borj-Cedria, BP 273, 8020, Soliman, University of Carthage, Tunisia.

[2]CNR-IPCF Sez. Bari, c/o Dept. of Chemistry, Università degli Studi di Bari, via Orabona 4, I-70126 Bari, Italy,

[3]Dept. of Chemistry, Università degli Studi di Bari, via Orabona 4, I-70126 Bari, Italy

Figure S1 reports the $(NH_4)_2MoS_4$ reduction current as a function of the precursor solution pH and time of the MoS_2 NRs electrodeposition by chronoamperometry.

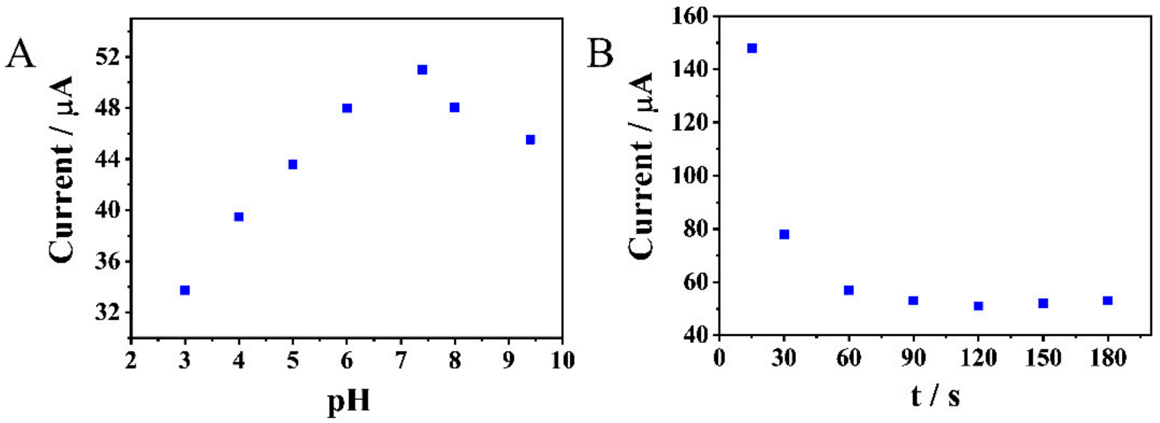

Figure S1. Reduction current of $(NH_4)_2MoS_4$ against (A) pH and (B) time of the chronoamperometry of the MoS_2 NRs, collected at – 1V (vs. Ag/AgCl, satured KCl) after dipping the PCA-rGO/PGEs in 5 mM $(NH_4)_2MoS_4$ aqueous solutions.

Figure S2 reports the SEM image of the neat PGEs and the TEM image of the PCA-rGO complex.

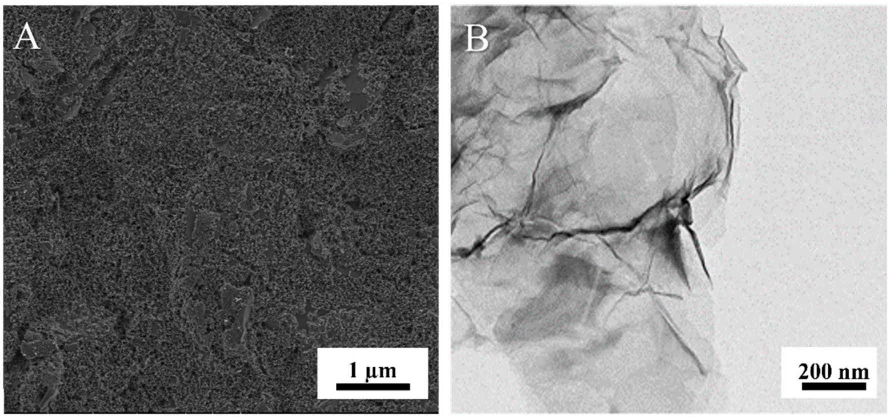

Figure S2. (A) SEM image of the bare PGEs (20 KX magnification) and (B) TEM micrograph of the PCA-rGO complex.

Figure S3A shows the current response at the MoS_2NRs/PCA-RGO/PGEs fabricated electrodepositing the MoS_2 NRs from $(NH_4)_2MoS_4$ solutions at pH ranging between 3-9.5, towards N_2H_4 and 4-NP solutions at pH 7.4, respectively. Figure 3B reports the current response at the MoS_2NRs/PCA-RGO/PGEs manufactured electrodepositing MoS_2 NRs from $(NH_4)_2MoS_4$ solutions at pH 7.4, towards N_2H_4 and 4-NP solutions, respectively at pH between 3-9.5.

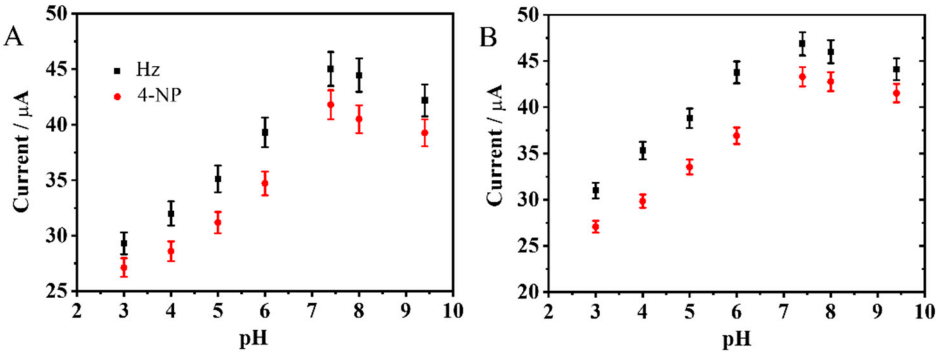

Figure S3. Oxidation and reduction currents of 0.4 mM N_2H_4 and 4-NP, respectively A) in solutions at pH 7.4, collected at the MoS_2NRs/PCA-RGO/PGEs manufactured electrodepositing $(NH_4)_2MoS_4$ from solutions at pH between 3-9.5, B) at pH between 3-9.5, recorded at the MoS_2NRs/PCA-RGO/PGEs fabricated electrodepositing $(NH4)_2MoS_4$ from solutions at pH 7.4.

Table S1. Comparative table of the LODs of N_2H_4 and 4-NP sensors reported in literature.

Electrode material	Target analyte	LOD (nM)	Reference
MoS_2/Au nanofoam/curcumin/PGE	N_2H_4	18.3	1
MoS_2/gold electrode		196	2
MoS_2/rGO/Au/GCE		500	3
MoS_2/GCE	4-NP	10	4
MoS_2/GCE		2.9	5
MoS_2/rGO/GCE		nd	6

Figure S4 reports nine chronoamperograms of N_2H_4 and 4-NP collected at the same MoS_2NRs/PCA-rGO/PGE in one day for repeatability tests.

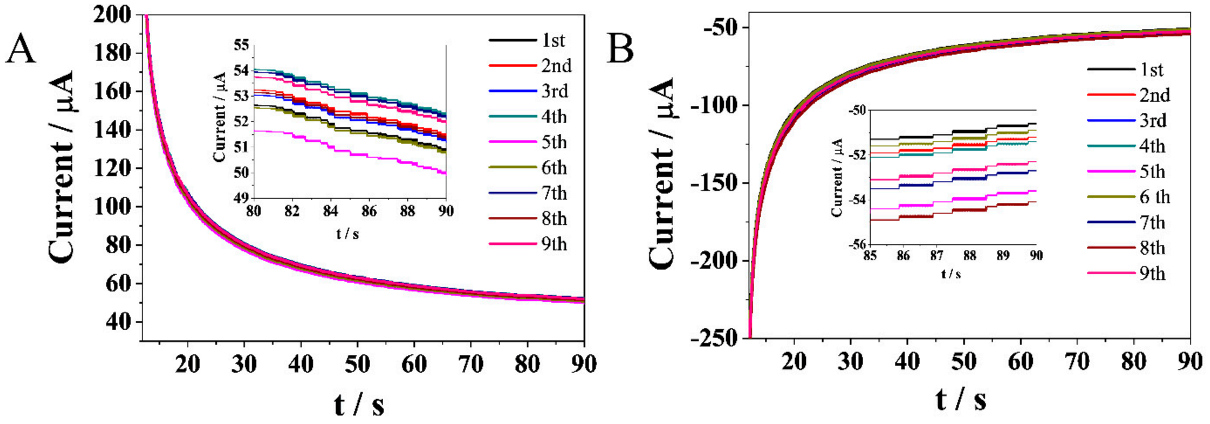

Figure S4. Chronoamperograms of repeatability tests recorded by using 0.5 mM N_2H_4 at +0.13 V (vs. Ag/AgCl, saturated KCl) (A) and 0.5 mM 4-NP at -0.71 V (B) (vs. Ag/AgCl, saturated KCl), collected at the same MoS_2NRs/PCA-rGO/PGE in 0.1 M PBS buffer solutions (pH 7.4).

Figure S5 reports the histograms of repeatability, reproducibility, storage stability and selectivity tests for the detection of N_2H_4 at the MoS_2NRs/PCA-rGO/PGEs.

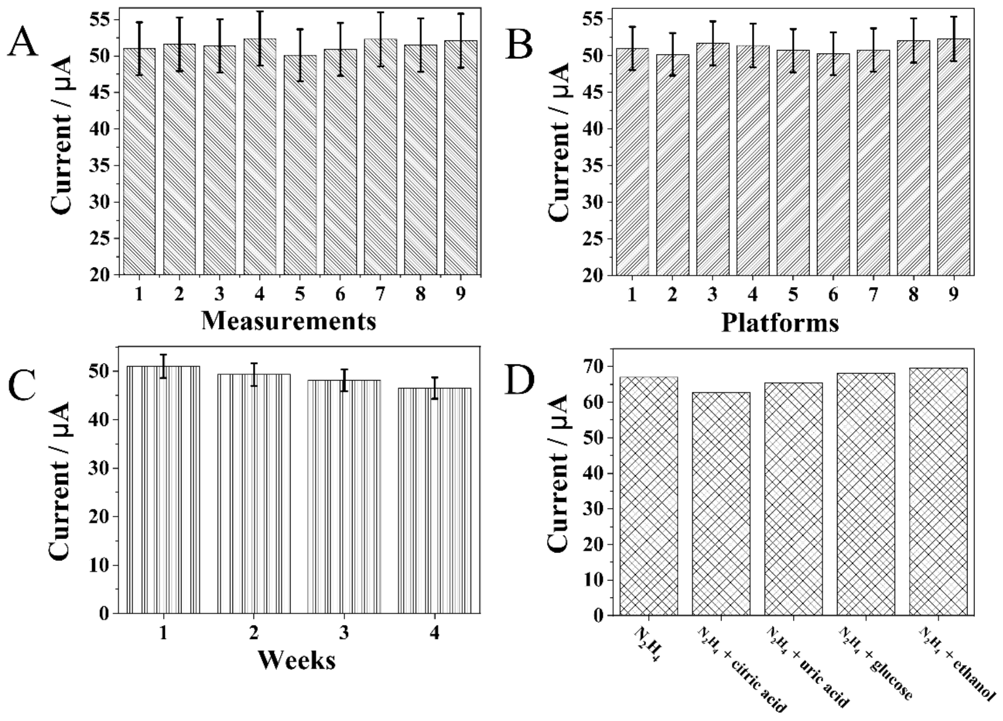

Figure S5. Histograms of repeatability (A), reproducibility (B), storage stability (C) and of selectivity tests (D) for the detection of N_2H_4 at the MoS_2NRs/PCA-rGO/PGEs.

Figure S6 reports the histograms of the repeatability, reproducibility, storage stability and selectivity tests for the detection of 4-NP at the MoS_2NRs/PCA-rGO/PGEs.

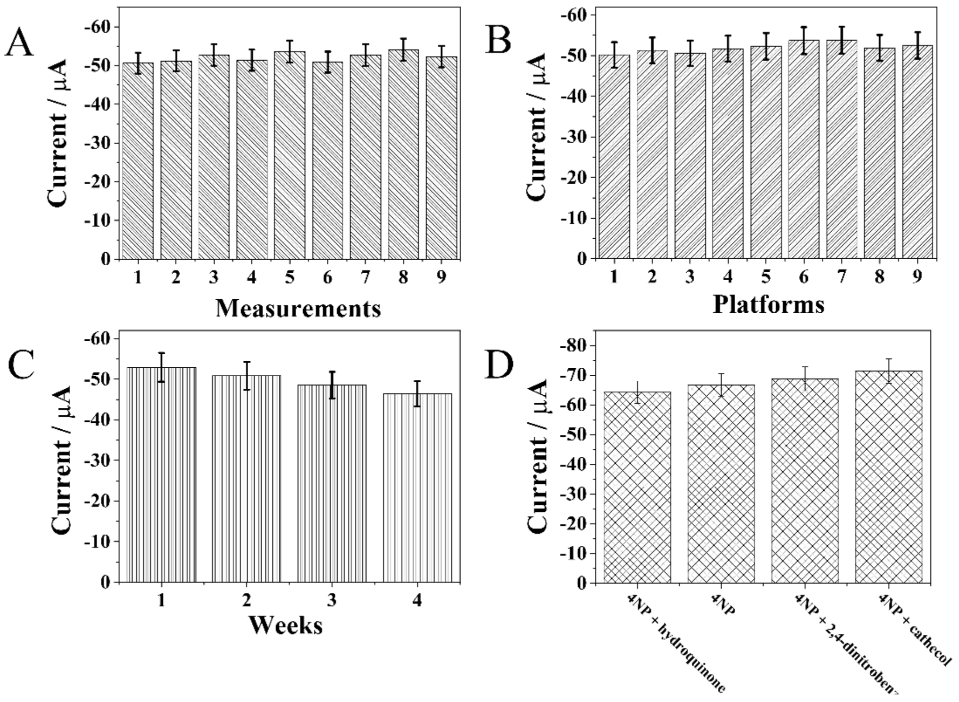

Figure S6. Histograms of repeatability (A), reproducibility (B), storage stability (C) and selectivity tests (D) for the detection of 4-NP at the MoS_2NRs/PCA-rGO/PGEs.

Figure S7 reports the chronoamperograms of N_2H_4 and 4-NP collected at nine MoS_2NRs/PCA-rGO/PGEs in one day for reproducibility tests.

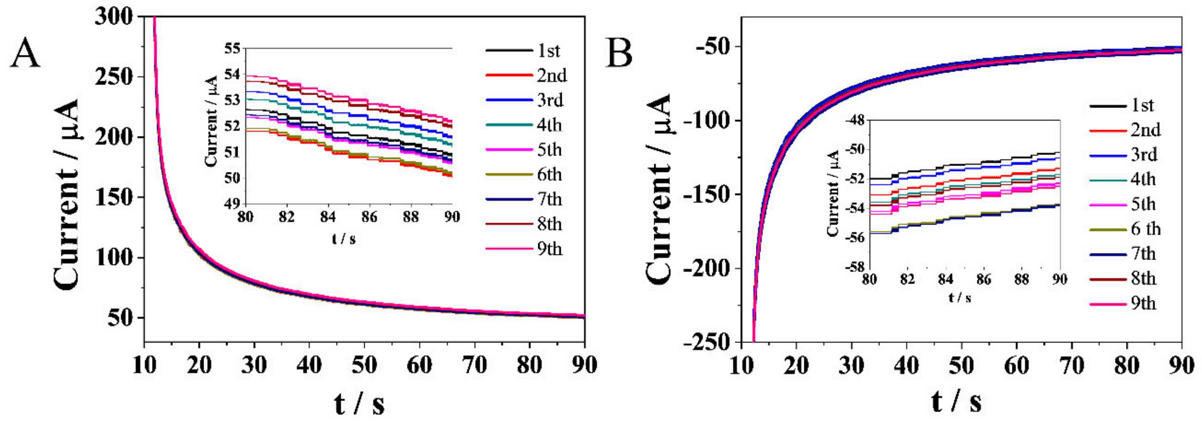

Figure S7. Chronoamperograms of reproducibility tests recorded by using 0.5 mM N_2H_4 at +0.13 V (vs. Ag/AgCl, saturated KCl) (A) and 0.5 mM 4-NP at -0.71 V (B) (vs. Ag/AgCl, saturated KCl), collected at the MoS_2NRs/PCA-rGO/PGEs in 0.1 M PBS buffer solutions (pH 7.4).

Figure S8 reports DPVs of N_2H_4 and 4-NP recorded at the MoS_2NRs/PCA-rGO/PGEs for storage stability tests.

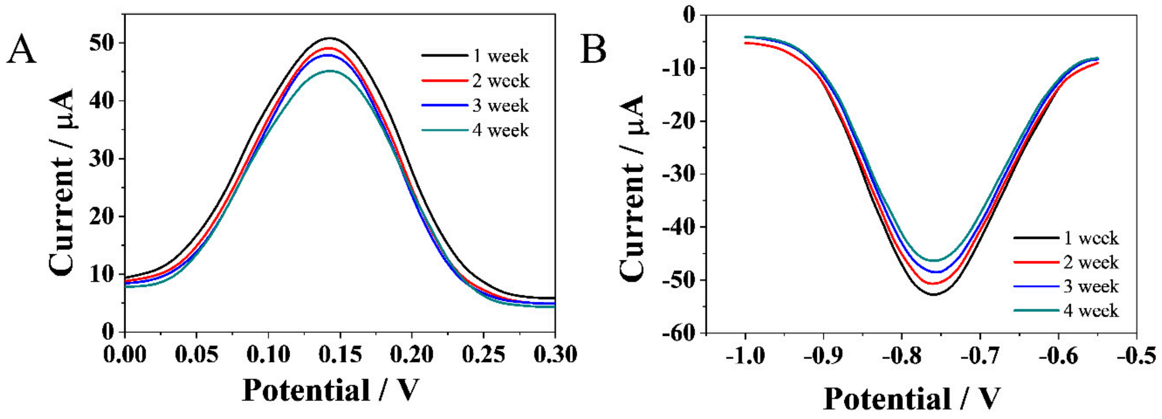

Figure S8. DPVs recorded at the MoS_2NRs/PCA-rGO/PGEs in 0.1 M PBS buffer (pH 7.4) added by 0.5 mM N_2H_4 (A) and 0.5 mM 4-NP (B), respectively, collected after one, two, three and four weeks with 0.05 s modulation time, 0.2 s interval time, 60 mV modulation amplitude, 10.5 mV step potential and 50 mV s^{-1} scan rate.

Figure S9 reports DPV curves of aqueous samples containing N_2H_4 and 4-NP, respectively, spiked with 100-folds higher concentrated interfering species.

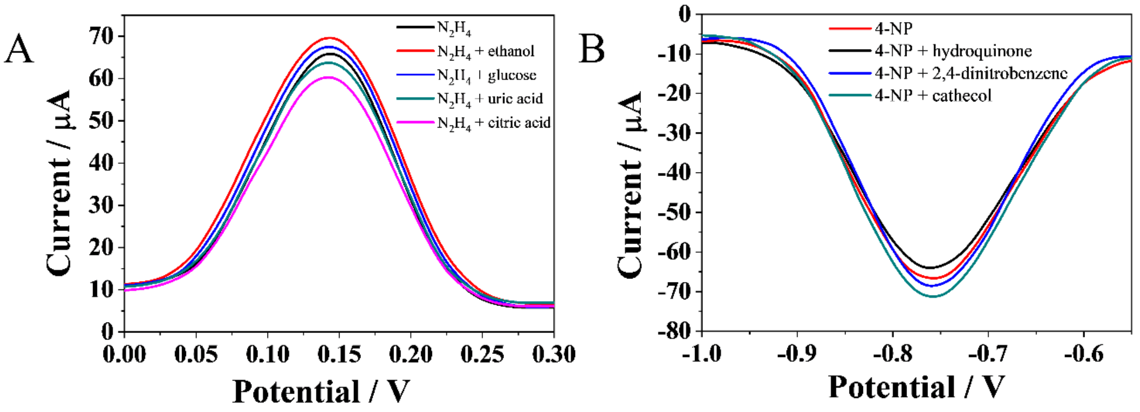

Figure S9. DPVs at the MoS_2NRs/PCA-rGO/PGEs in 0.1 M PBS buffer (pH 7.4) added by 0.8 mM N_2H_4 (A) and 0.8 mM 4-NP (B), respectively in presence of 80 mM interfering species, with 0.05 s modulation time, 0.2 s interval time, 60 mV modulation amplitude, 10.5 mV step potential and 50 mV s^{-1} scan rate.

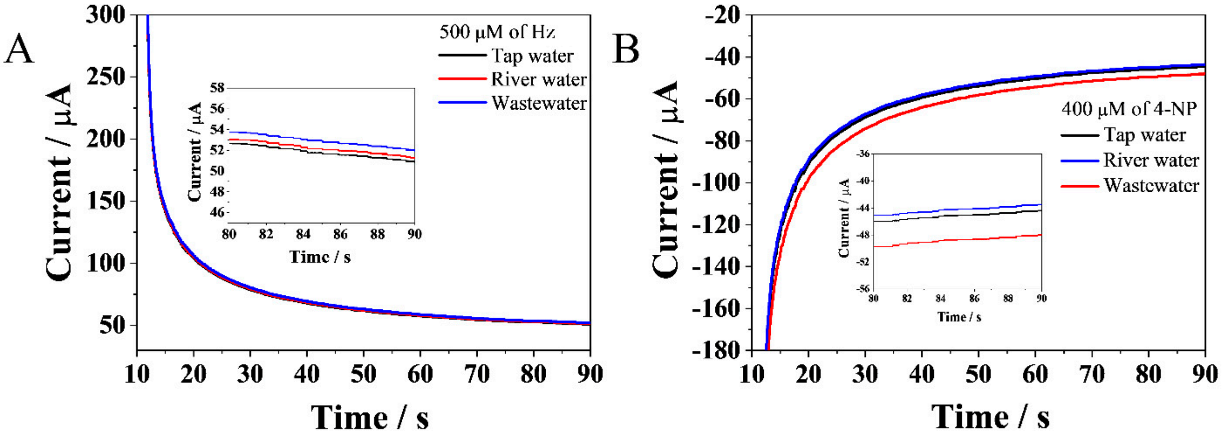

Figure S10. Chronoamperograms at the MoS_2NRs/PCA-rGO/PGEs recorded in the detection of (A) 500 µM N_2H_4 and (B) 400 µM 4-NP in tap, river, and wastewater samples, at 0.13 V and -0.71 V (vs. Ag/AgCl, saturated KCl), respectively.

References

1. Mejri, A.; Mars, A.; Elfil, H.; Hamzaoui, A. H. Curcumin graphite pencil electrode modified with molybdenum disulfide nanosheets decorated gold foams for simultaneous quantification of nitrite and hydrazine in water samples. *Anal. Chim. Acta.* **2020**, *1137*, 19-27.
2. Rana, D. S.; Thakur, N.; Thakur, S.; Singh, D. Electrochemical determination of hydrazine by using MoS_2 nanostructure modified gold electrode. *Nanofabrication* **2021**, 6, e002.
3. Gharani, M.; Bahari, A.; Ghasemi, S. Preparation of MoS_2-reduced graphene oxide/Au nanohybrid for electrochemical sensing of hydrazine, *J Mater Sci: Mater Electron.* **2021**, *32*, *7765–7777.*
4. Jeyapragasam, T.; Devi, M.; Ganesh, V. Molybdenum disulfide-based modifier for electrochemical detection of 4-nitrophenol. *Ionics* **2018**, *24*, 4033–4041.
5. Nehru, R.; Kumar, B. S.; Chen, C. W.; Di-Dong C. Yolk-shell structured molybdenum disulfide nanospheres as highly enhanced electrocatalyst for electrochemical sensing of hazardous 4-nitrophenol in water. *Journal of Environmental Chemical Engineering* **2022**, *10*, 107663.
6. Chen, Y.; Peng, W. Synthesis of MoS2/Graphene Hybrid for Electrochemical Detection and Catalytic Reduction of 4-Nitrophenol. *Applied Mechanics and Materials* **2017**, *872*, 149-154.
